# Supplementary material for: Freshwater carbon and nutrient cycles revealed through reconstructed population genomes
Source: PeerJ. 2018 Dec 10;6:e6075. doi: 10.7717/peerj.6075 (PMC6292386; doi:10.7717/peerj.6075)
Supplement: Supplemental Information 1 — Additional chemistry data were collected by NTL-LTER (http://lter.limnology.wisc.edu) from depth discrete samples taken from 0 and 4 m for Mendota, 0 m for the Trout Bog epilimnion, and 3 and 7 m for the Trout Bog hypolimnion. Values reported here are the means of all measurements in the sampling time span for each lake, with standard deviations reported in parentheses. [file peerj-06-6075-s001.docx]

**Table S1. Additional chemical concentrations in our study sites.** Additional chemistry data was collected by NTL-LTER (<http://lter.limnology.wisc.edu>) from depth discrete samples taken from 0 and 4 m for Lake Mendota, 0 m for the Trout Bog Epilimnion, and 3 and 7 m for the Trout Bog Hypolimnion. Values reported here are the means of all measurements in the sampling time span for each lake, with standard deviations reported in parentheses.

|  | Lake Mendota | Trout Bog Epilimnion | Trout Bog Hypolimnion |
| --- | --- | --- | --- |
| Chloride (ppm) | 44.5 (4.5) | 0.24 (0.08) | 0.29 (0.09) |
| Calcium (ppm) | 30.0 (4.9) | 1.29 (0.48) | 1.88 (0.37) |
| Magnesium (ppm) | 31.2 (2.5) | 0.37 (0.13) | 0.47 (0.07) |
| Sodium (ppm) | 20.5 (2.2) | 0.23 (0.09) | 0.27 (0.06) |
| Potassium (ppm) | 3.1 (0.3) | 0.6 (0.3) | 0.72 (0.20) |
| Iron (ppm) | 0.01 (0.01) | 0.29 (0.15) | 0.47 (0.10) |
| Manganese (ppm) | 0.01 (0.02) | 0.08 (0.08) | 0.10 (0.14) |
