# Supplementary material for: Freshwater carbon and nutrient cycles revealed through reconstructed population genomes
Source: PeerJ. 2018 Dec 10;6:e6075. doi: 10.7717/peerj.6075 (PMC6292386; doi:10.7717/peerj.6075)
Supplement: Supplemental Information 2 — Metagenomic samples were pooled by lake and layer to allow time-resolved binning. The Mendota time series spans 2008–2012, while the Trout Bog time series spans 2007–2009. Just under 200 medium to high quality metagenome-assembled genomes (MAGs) were produced. [file peerj-06-6075-s002.docx]

**Table S2. Statistics from genome assembly and binning.** Metagenomic samples were pooled by lake and layer to allow time-resolved binning. The Lake Mendota time series spans 2008-2012, while the Trout Bog time series spans 2007-2009. Just under 200 medium to high quality metagenome-assembled genomes (MAGs) were produced.

| IMG Genome ID | 3300002835 | 3300000439 | 3300000553 |
| --- | --- | --- | --- |
| Lake-layer | Lake Mendota | Trout Bog Epilimnion | Trout Bog Hypolimnion |
| Number of metagenomes | 94 | 47 | 47 |
| Collection time span | Jun. 2008 – Nov. 2012 | Jun. 2007 – Aug. 2009 | May 2007 – Aug. 2009 |
| Number of metagenomes per year | 2008:16, 2009:15, 2010:21, 2011:14, 2012:28 | 2007:15, 2008:18, 2009:14 | 2007:17, 2008:16, 2009:14 |
| Total base pairs in metagenomes | 1.26x10^11^ | 6.72x10^10^ | 7.18x10^10^ |
| Merged read length quartiles (subsample of 10,000 reads per metagenome) | 0%: 151, 25%: 194, 50%: 218, 75%: 242, 100%: 292 | 0%: 151, 25%: 188, 50%: 214, 75%: 242, 100%: 290 | 0%: 151, 25%: 187, 50%: 212, 75%: 241, 100%: 290 |
| Total base pairs in pooled assembly | 3.37x10^9^ | 2.60x10^8^ | 5.47x10^8^ |
| Number of contigs in pooled assembly | 9,912,431 | 79,862 | 153,912 |
| Number of curated bins | 99 | 31 | 63 |
| Number of base pairs in curated bins | 2.31x10^8^ | 5.82x10^7^ | 1.60x10^8^ |
| Number of contigs in curated bins | 18,675 | 5,098 | 11,656 |
