# Supplementary material for: Freshwater carbon and nutrient cycles revealed through reconstructed population genomes
Source: PeerJ. 2018 Dec 10;6:e6075. doi: 10.7717/peerj.6075 (PMC6292386; doi:10.7717/peerj.6075)
Supplement: Supplemental Information 3 — A Wilcoxon rank sum test was used to non-parametrically test for significant differences in functional marker gene distributions between our study sites. P-values of less than 0.05 are considered significant. [file peerj-06-6075-s003.docx]

| Functional Marker | Mendota vs Trout Bog Epilimnion | Trout Bog Epilimnion vs Hypolimnion |
| --- | --- | --- |
| RubisCO | 0* | 0* |
| Urease | 0.69 | 1 |
| Nitrogenase | 0* | 0* |
| Nitrate reductase | 1 | 0* |
| Nitrite reductase | 0* | 0* |
| Nitric oxide reductase | 0.83 | 0* |
| Nitrous oxide reductase | 0* | 0.01* |
| SOX | 0* | 0.04* |
| Sulfate adenylyltransferase | 0.94 | 0* |
| Sulfide quinone reductase | 0* | 0* |
| Sulfite reductase | 1 | 0* |
| FeFe hydrogenase | 0.26 | 0* |
| [Ni-Fe] hydrogenase group 1 | 0.03* | 0* |
| [Ni-Fe] hydrogenase group 2a | 1 | 0* |
| [Ni-Fe] hydrogenase group 2b | 0.03* | 1 |
| [Ni-Fe] hydrogenase group 3a | 0* | 1 |
| [Ni-Fe] hydrogenase group 3b | 0.2 | 1 |
| [Ni-Fe] hydrogenase group 3c | 1 | 0* |
| [Ni-Fe] hydrogenase group 3d | 0.07* | 0* |
| [Ni-Fe] hydrogenase group 4 | 1 | 1 |

**Table S3. P-values of marker gene distributions between sites.** A Wilcoxon rank sum test was used to non-parametrically test for significant differences in functional marker gene distributions between our study sites. P-values of less than 0.05 are considered significant.
