# Supplementary figures and images for: Freshwater carbon and nutrient cycles revealed through reconstructed population genomes
Source: PeerJ. 2018 Dec 10;6:e6075. doi: 10.7717/peerj.6075 (PMC6292386; doi:10.7717/peerj.6075)

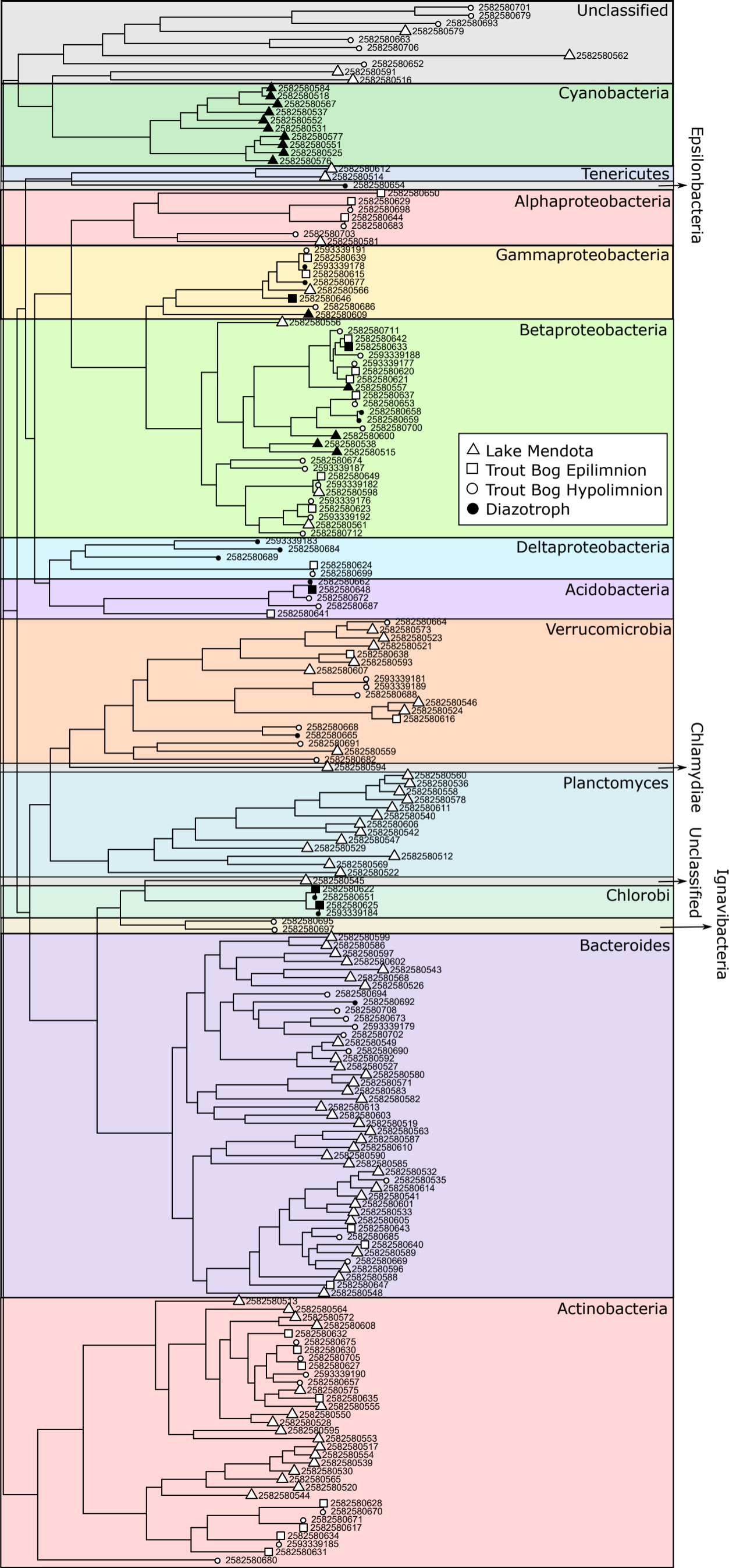

Supplement: Supplemental Information 10 — To visualize the diversity of our MAGs, phylogenetic marker genes were extracted from each MAG and aligned using Phylosift. An approximate maximum-likelihood tree based on these alignments was constructed using FastTree. The potential for nitrogen fixation based on gene content is indicated on the branch tips. [file peerj-06-6075-s010.pdf]

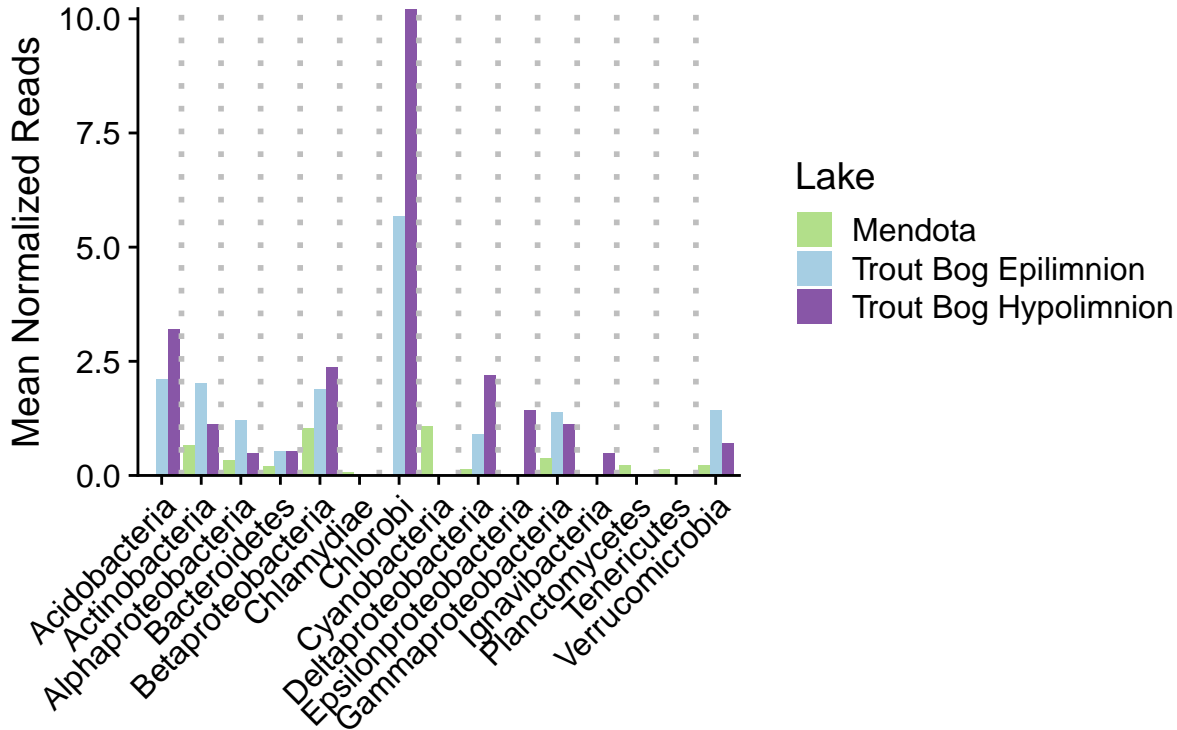

Supplement: Supplemental Information 11 — We used read coverage normalized by MAG and metagenome size to approximate the abundance of our MAGs. MAGs were recovered from diverse freshwater phyla. The abundances of phyla represented by MAGs differed by lake and layer. MAGs were classified using Phylosift, and Proteobacteria was split into classes due to the high diversity of this phylum. [file peerj-06-6075-s011.pdf]

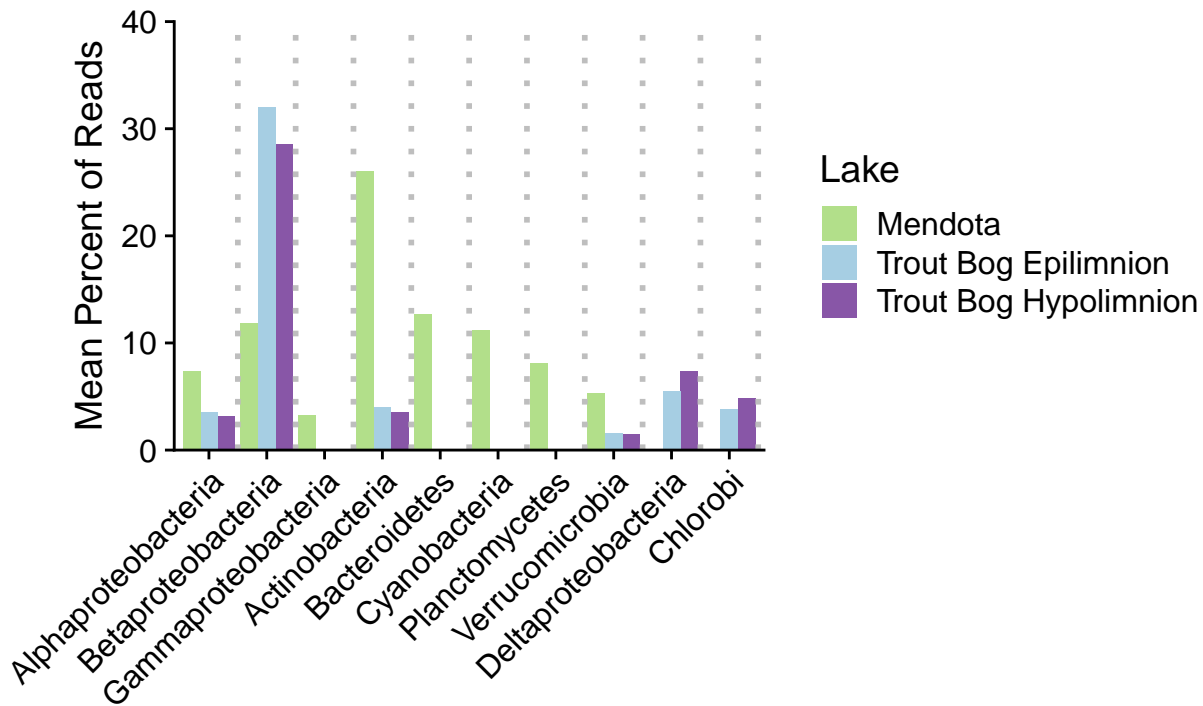

Supplement: Supplemental Information 12 — The community composition observed via 16S rRNA gene amplicon sequencing in our dataset is consistent with previously published analyses of freshwater community composition. This confirms that the years included in our study are not abnormal. The 16S V6–V8 region was targeted in Trout Bog, while the V4 region was targeted in Mendota. Proteobacteria was split into classes due to the high diversity of this phylum. [file peerj-06-6075-s012.pdf]
